# Cost-Effectiveness of Apatinib and Cabozantinib for the Treatment of Radioiodine-Refractory Differentiated Thyroid Cancer

**DOI:** 10.3389/fphar.2022.860615

**Published:** 2022-06-30

**Authors:** Bo Shi, Wenbiao Ma, Hongshuai Pan, Yang Shi, Huan Zhang, Shenghai Xing

**Affiliations:** ^1^ Department of Breast and Thyroid Surgery, People’s Hospital of Qinghai Province, Xining, China; ^2^ Department of General Surgery, People’s Hospital of Qinghai Province, Xining, China; ^3^ Department of Gynecology, Second Beijing Hospital, Beijing, China; ^4^ Department of Anesthesiology, People’s Hospital of Qinghai Province, Xining, China; ^5^ Department of Nuclear Medicine, People’s Hospital of Qinghai Province, Xining, China

**Keywords:** apatinib, cabozantinib, radioactive iodine-refractory differentiated thyroid cancer, cost-effective, China

## Abstract

**Background:** The effectiveness of apatinib and cabozantinib for the treatment of radioactive iodine–refractory differentiated thyroid cancer (RAIR-DTC) has been demonstrated recently. We aimed to evaluate the cost-effectiveness of these treatments from the Chinese healthcare system perspective.

**Methods:** Two partitioned survival models over a 10-year horizon were built to compare the cost and effectiveness of apatinib vs. placebo and cabozantinib vs. placebo based on the clinical data from the phase 3 randomized REALITY and COSMIC-311 trials. Costs and utility data were obtained from the literature and institutional database. The incremental cost-effectiveness ratio (ICER) was calculated. One-way and probabilistic sensitivity analysis was performed to test the robustness of the conclusion.

**Results:** Apatinib yielded an additional quality-adjusted life-year (QALY) of 0.74 at an additional cost of Chinese Renminbi ¥44,077. The ICER was ¥93,460 (US dollar $13545)/QALY and it was below the current willingness-to-pay (WTP) threshold of ¥217341/QALY. Cabozantinib was associated with an additional QALY of 0.79 at an extra cost of ¥3,55,614 when compared with placebo, and the ICER was ¥4,52,325 ($65,554)/QALY, which was above the WTP threshold. The conclusion were robust under one-way and probabilistic sensitivity analysis. The price of cabozantinib has to drop to ¥5.87/mg (39% of the current price) for it has a 50% likelihood of being cost-effective.

**Conclusion:** Apatinib is cost-effective for RAIR-DTC when compared with placebo from the perspective of Chinese healthcare system. However, based on the current evidence, cabozantinib might not be cost-effective and a reduction of price is warranted.

## Introduction

Thyroid cancer is a common endocrine malignancy with an estimated 5,86,202 new cases in 2020 globally and China has near 1/3 of all these incidence cases worldwide (Siegel et al., 2016; Sung et al., 2021). Differentiated thyroid cancer (DTC) is the most common type, accounting for 90%–95% of newly diagnosed thyroid cancers, and is composed of papillary (80%), follicular (10%–15%), less frequent Hürthle cell, and poorly differentiated cancers (5%–10%) (Schlumberger and Leboulleux, 2021). The prognosis of DTC is relatively favorable after treatment including active surveillance, surgery, or radioiodine therapy. However, up to 15% of patients might become refractory to radioactive iodinetherapy and have a poor prognosis (Durante et al., 2006; Lirov et al., 2017). Treatment options for radioactive iodine–refractory DTC (RAIR-DTC) include angiogenesis inhibitors sorafenib and lenvatinib, which were found to prolong progression-free survival (PFS) (Brose et al., 2014; Lee et al., 2015). For patients with RAIR-DTC that initially achieved disease control with sorafenib or lenvatinib, a relatively high proportion of them will eventually develop treatment resistance and lead to poor prognosis (Brose et al., 2014; Lee et al., 2015). The unmet clinical needs for RAIR-DTC warrant the exploration of alternative strategies.

Recently, two randomized phase 3 trials have assessed the efficacy and safety of apatinib (the REALITY trial, NCT03048877) and cabozantinib (the COSMIC-311 trial, NCT03690388), the highly selective vascular endothelial growth factor inhibitors, in patients with RAIR-DTC (Brose et al., 2021; Lin et al., 2021). The REALITY trial showed that apatinib could prolong both PFS and overall survival (OS) with a manageable safety profile and the COSMIC-311 trial demonstrated that cabozantinib significantly prolongs PFS while the median OS did not reach data cutoff. Based on these results, the United States Food and Drug Administration approved cabozantinib for locally advanced or metastatic RAIR-DTC on 17 September 2021 (United States Food and Drug Administration, 2021). Therefore, apatinib and cabozantinib might provide new treatment options for these patients.

Physicians and practice settings sometimes need to evaluate the cost-efficiency of the new therapies compared with the alternatives, especially in some resource-limited countries like China as the new therapies are associated with significantly higher costs. The best way of doing this is through cost-effectiveness analysis. It estimates the incremental cost-effectiveness ratio (ICER) of a new treatment versus the old treatment by calculating the ratio of additional costs over the additional quality-adjusted life-years (QALYs). Currently, there lacks evidence on the economic value of these two drugs. In this study, we aimed to evaluate the cost-effectiveness of apatinib and cabozantinib vs. placebo for the treatment of RAIR-DTC from the Chinses healthcare system perspective.

## Methods

### Patients and Intervention

This cost-effectiveness analysis was based on a literature review and modeling techniques. Approval from the Institutional Review Board of our hospital was exempted because no real human participants were involved. The target population in this study was based on the REALITY and COSMIC-311 trials: age 16 years or older, locally advanced or metastatic RAIR-DTC that progressed within 12 months after the last treatment, and a life expectancy of 3 months or more.

Included patients received 500 mg apatinib (60 mg cabozantinib) or matching placebo tablets orally once daily until disease progression. During the extension phase after disease progressed, patients would receive apatinib or cabozantinib openly in both apatinib (or cabozantinib) and placebo groups at the discretion of the investigators.

### Model Construction

We built two partitioned survival (PS) models with TreeAge Pro 2020 (TreeAge, Williamstown, MA) to compare costs and clinical outcomes associated with apatinib or cabozantinib vs. placebo for the treatment of RAIR-DTC. The PS model is frequently utilized in oncology modeling and the proportion of patients in different health states including progression-free disease state (PFD), progressed disease state (PD), and death state at different time points was derived from PFS and OS curves directly (Glasziou et al., 1990). The cycle length in our models was 4 weeks, which was similar to the REALITY and COSMIC-311 trials. The time horizon was 10 years in which 95% of patients would be dead in both treatment arms. This study was conducted from the Chinese healthcare system perspective. A 5% discount rate per year was applied for both cost and effectiveness according to the China Guidelines for Pharmacoeconomic Evaluation (Chinese Pharmaceutical Association, 2021). The willingness-to-pay threshold (WTP) of Chinese Renminbi ¥72,447 to ¥2,17,341 per QALY gained (1–3 times of gross domestic product per capita) was used in China (National Bureau of Statistics of China, 2021).

### Clinical Data Inputs

**FIGURE 1:**
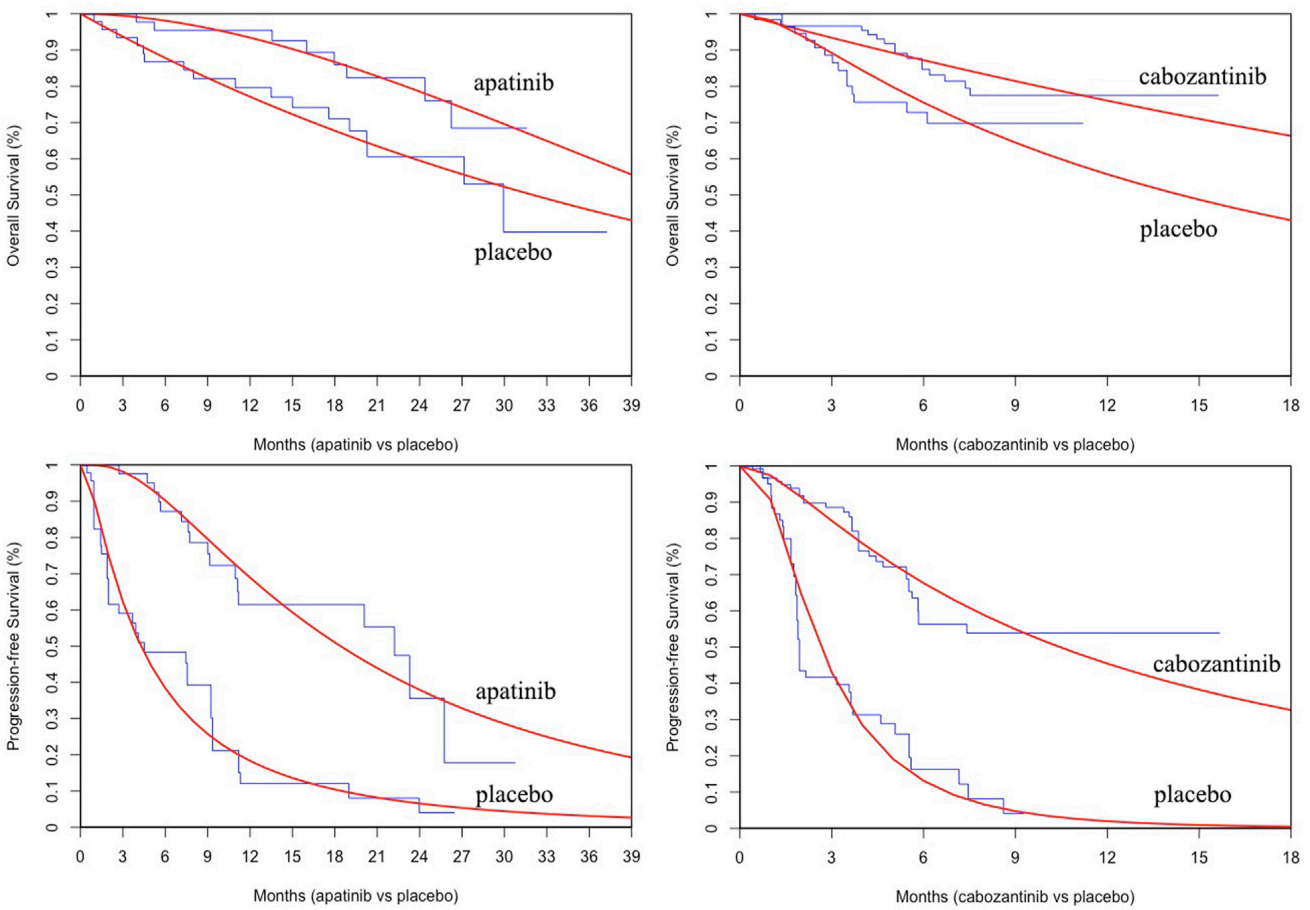
The comparison between reconstructed Kaplan-Meier curves from the REALITY and COSMIC-311 trials and the best parametric fitting curves.

PFS and OS curves from the REALITY and COSMIC-311 trials were modeled according to the methods described by Guyot et al. (2012) and Baio (2020). In brief, we first collected data points from the published PFS and OS curves by using the GetData Graph Digitizer (Version 2.26, https://getdata-graph-digitizer.com/) and reconstrued these curves with the generated pseudoindividual patient data. Then the reconstructed curves were used to fit the following survival functions including gompertz, exponential, gamma, genf, gengamma, weibull, weibullPH, loglogistic, and lognormal. The best-fit survival function was determined by the smallest Akaike information criterion and Bayesian information criterion (Latimer, 2013). The parameters of best-fitting functions for PFS and OS curves are provided in Table 1 and the comparison between reconstructed and parametric fitting curves is presented in Figure 1.

**TABLE 1:** Basic parameters input to the model and the ranges for sensitivity analyses.

Parameters	Expected value	Range	Distribution	Source
Clinical Data				
Weibull OS survival model of apatinib in the REALITY trial	Shape: 1.827	—	—	Model fitting
	Scale: 52.206			
Exponential OS survival model of placebo the REALITY trial	Rate: 0.02167	—	—	Model fitting
Lognormal PFS survival model of apatinib the REALITY trial	Meanlog: 2.912	—	—	Model fitting
	Sdlog: 0.866			
Lognormal PFS survival model of placebo the REALITY trial	Meanlog: 1.454	—	—	Model fitting
	Sdlog: 1.142			
Exponential OS survival model of cab in the COSMIC-311 trial	Rate: 0.02285	—	—	Model fitting
Gengamma OS survival model of placebo in the COSMIC-311 trial	Mu: 0.3302		—	Model fitting
	Sigma: 0.1197			
	Q: −41.8983			
Lognormal PFS survival model of cabozantinib in the COSMIC-311 trial	Meanlog: 2.346	—	—	Model fitting
	Sdlog: 1.209			
Gengamma PFS survival model of placebo in the COSMIC-311 trial	Mu: 0.5973		—	Model fitting
	Sigma: 0.6428			
	Q: −1.1205			
Cost (Chinese Yuan Renminbi, ¥)				
Apartinib	70 per 250 mg	49–91	Gamma, SD: 10.5	Tuling
Cabozantinib	15.03 per mg	10.52–19.54	Gamma, SD: 2.25	Liao et al. (2019)
Best-supportive care per cycle	4,000	2,800–5,200	Gamma, SD: 600	Hospital charges
Follow-up per two cycles during the first year	1,300	910–1,690	Gamma, SD: 195	Hospital charges
Follow-up per three cycles after the first year	1,050	735–1,365	Gamma, SD: 158	Hospital charges
End-of-life care per cycle	8,500	5,950–11,050	Gamma, SD: 1,275	Hospital charges
Management of severe AE				
Hypertension	7,326	5,128–9,524	Gamma, SD: 1,099	Hospital charges
Hand-foot syndrome	8,455	5,919–10,992	Gamma, SD: 1,268	Hospital charges
Proteinuria	1,174	822–1,526	Gamma, SD: 176	Hospital charges
Diarrhea	4,150	2,905–5,395	Gamma, SD: 623	Hospital charges
Hypocalcaemia	2,445	1712–3,179	Gamma, SD: 367	Hospital charges
Deep vein thrombolysis	3,723	2,606–4,840	Gamma, SD: 558	Hospital charges
Pulmonary embolism	14,242	9,969–18,515	Gamma, SD: 2,136	Hospital charges
Probability of treatment-related severe AE among patients with apatinib				
Hypertension	0.348	0.227–0.492	Gamma, SD: 0.066	Lin et al. (2021)
Hand-foot syndrome	0.174	0.091–0.307	Gamma, SD: 0.054	Lin et al. (2021)
Proteinuria	0.152	0.076–0.282	Gamma, SD: 0.052	Lin et al. (2021)
Diarrhea	0.152	0.076–0.282	Gamma, SD: 0.052	Lin et al. (2021)
Hypocalcaemia	0.065	0.022–0.175	Gamma, SD: 0.038	Lin et al. (2021)
Probability of treatment-related severe AE among patients with cabozantinib				
Diarrhea	0.032	0.013–0.079	Gamma, SD: 0.017	Brose et al. (2021)
Deep vein thrombolysis	0.008	0.001–0.044	Gamma, SD: 0.011	Brose et al. (2021)
Hypertension	0.016	0.004–0.057	Gamma, SD: 0.013	Brose et al. (2021)
Pulmonary embolism	0.016	0.004–0.057	Gamma, SD: 0.013	Brose et al. (2021)
Utility				
Progression-free disease	0.80	0.77–0.84	Beta, SD: 0.018	Fordham et al. (2015)
Progression of the disease	0.50	0.45–0.56	Beta, SD: 0.028	Fordham et al. (2015)
Disutility due to severe AE	0.25	0.21–0.35	Beta, SD: 0.035	Fordham et al. (2015)

AE, adverse events; OS, overall survival; PFS, progression-free survival; SD, standard deviation.

### Costs

Only direct costs were considered in this study, including costs for drugs, follow-up, severe treatment-related adverse events management, best-supportive care, and end-of-life care. The unite price of apatinib was based on the median price from the popular Chinese drug price database (Tuling, www.315jiage.cn). We compared the price of apatinib from this database with other famous Chinese online pharmacies as well as our institutional clinical database and these prices were very close. Since cabozantinib is not available on the mainland Chinese market, we obtained its unit price from the literature in which the authors used the Hongkong price to investigate the cost-effectiveness of cabozantinib as second-line therapy in advanced hepatocellular carcinoma from a Chinese perspective (Liao et al., 2019). The cost for follow-up, adverse events management, best-supportive care, and end-of-life care was calculated from our institutional database. According to the COSMIC-311 trial, patients were assumed to have continuous best-supportive care when they entered the model. They would also have end-of-life care after disease progressed. We compared our prices with those from other similar cost-effectiveness studies conducted in China and the differences were moderate (Dranitsaris et al., 2015; Zhang et al., 2019; Chen et al., 2021). Moreover, to make sure our prices were representative enough and account for uncertainties, we used a wide range of ±30% in the sensitivity analyses. All costs were converted to 2020 values according to the local Consumer Price Index if necessary.

### Utilities

Since the quality-of-life data was not reported by the REALITY nor COSMIC-311 trial, we assigned a value of 0.8 for PFD and 0.5 for PD according to the study by Fordham et al. (2015) in which the health state utility valuation in RAIR-DTC was elicited. Patients with severe adverse events were assumed to have a disutility of 0.25 and all these events were assumed to incur in the first cycle for the convenience of calculation.

### Statistical Analysis

Overall costs, drug costs, life-years, QALYs, and ICER were calculated in the base-case analysis. One-way sensitivity and probabilistic sensitivity analysis were also performed to assess the robustness of the model. The ranges used in the one-way sensitivity analysis were based on either the reported 95% confidence interval in the literature or a ±30% change from the base value. In the probabilistic sensitivity analysis with a Monte Carlo simulation (1,000 iterations), all variables were sampled simultaneously within their prespecified distributions in which costs were assigned with a gamma distribution while probability and utilities were assigned with a beta distribution. A cost-effectiveness acceptability curve was generated based on the results of 1,000 iterations to evaluate the likelihood of apatinib or cabozantinib being cost-effective at different WTP thresholds.

## Results

### Base Case Results

In the base case analysis (Table 2), when compared with placebo, apatinib had an additional cost of ¥44,077 with an additional QALY of 0.44 and the ICER was ¥93,460 (US dollar $13,545) per QALY. Cabozantinib yielded an additional 0.79 QALY at an additional cost of ¥3,55,614 when compared with placebo. The ICER was ¥4,52,325 ($65,554)/QALY.

**TABLE 2:** Results of base-case analysis.

	Apatinib			Cabozantinib		
	Apatinib	Placebo	Differences	Cabozantinib	Placebo	Differences
Overall costs (¥)	3,05,742	2,64,748	44,077	6,30,129	2,74,515	3,55,614
Drug costs (¥)	1,09,977	88,653	21,323	4,62,740	1,59,627	3,03,133
Life-years	3.19	2.88	0.30	2.77	1.85	0.92
QALYs	1.92	1.48	0.44	1.78	1.00	0.79
ICER (¥/QALY)			93,460			4,52,325

ICER, incremental cost-effectiveness ratio; QALYs, quality-adjusted life-years.

### One-Way Sensitivity Analysis

**FIGURE 2:**
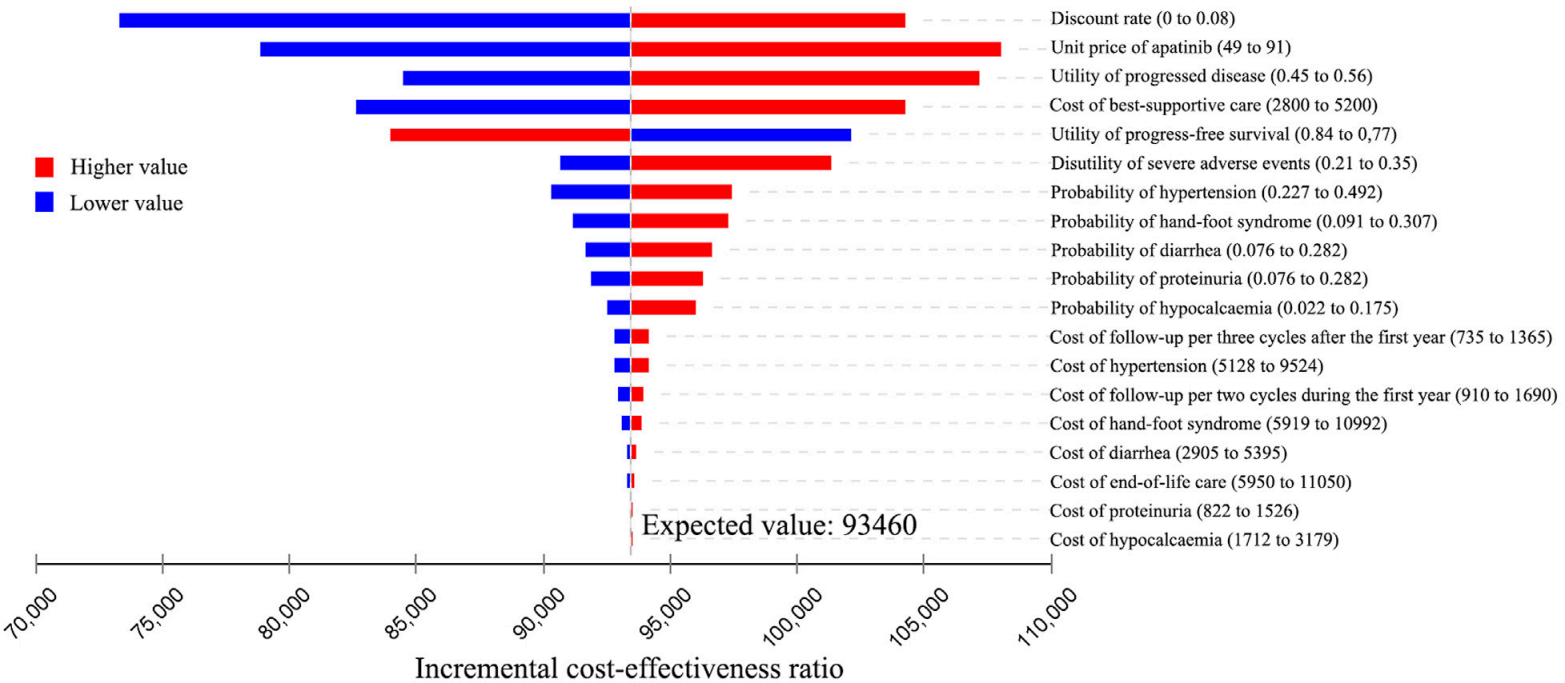
Tornado diagram of one-way sensitivity analyses of apatinib vs. placebo for the treatment of radioactive iodine–refractory differentiated thyroid cancer.

**FIGURE 3:**
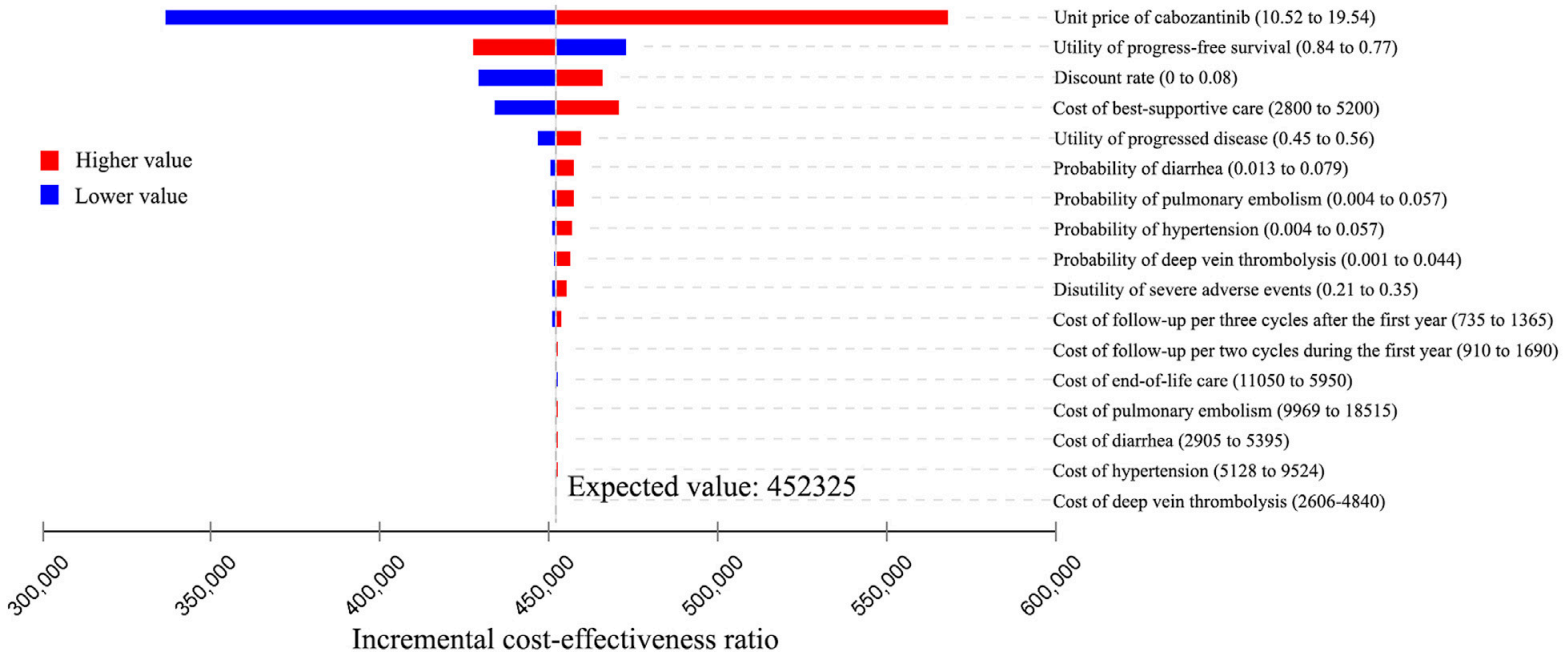
Tornado diagram of one-way sensitivity analyses of cabozantinib vs. placebo for the treatment of radioactive iodine–refractory differentiated thyroid cancer.

The results of the one-way sensitivity analysis were presented in Tornado diagrams, showing the effect of uncertainty in each parameter on the ICER. For patients treated with apatinib, the results were more sensitive to the discount rate, unit price of apatinib, utility of PD, cost of best-supportive care, and utility of PFS (Figure 2). Across the broad variation in the ranges of these parameters, the ICERs were all below the current Chinese WTP threshold of ¥2,17,341 per QALY gained. For patients treated with cabozantinib, the results were more sensitive to the unit price of cabozantinib, utility of PFS, discount rate, and cost of best-supportive care (Figure 3). All the ICERs were above the WTP threshold when these parameters varied in their ranges.

### Probabilistic Sensitivity Analysis

**FIGURE 4:**
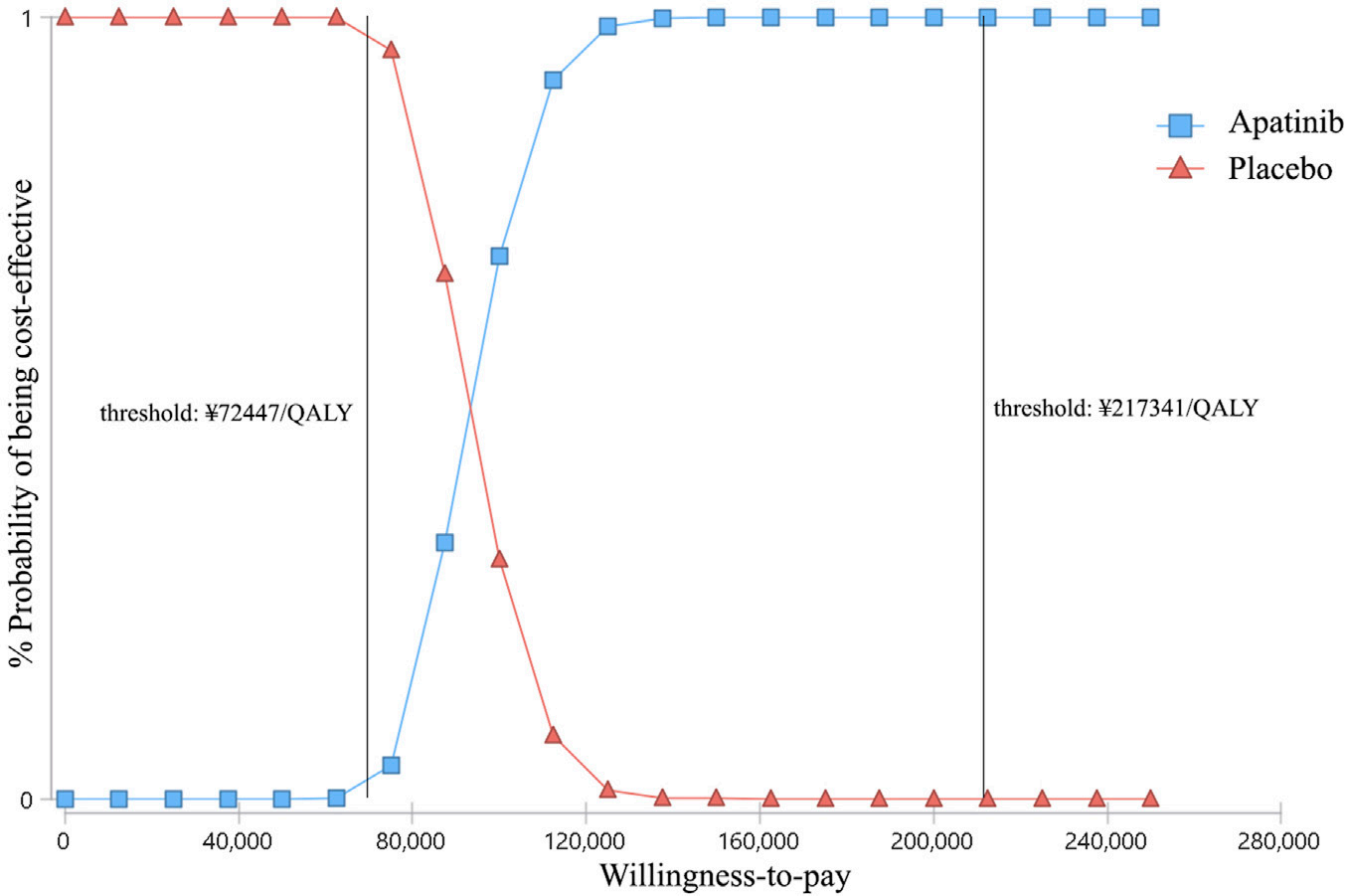
Cost-effectiveness acceptability curves of apatinib versus placebo for the treatment of radioactive iodine–refractory differentiated thyroid cancer. The square shapes indicate the probability of apatinib being cost-effective when compared with placebo under different willingness-to-pay thresholds.

**FIGURE 5:**
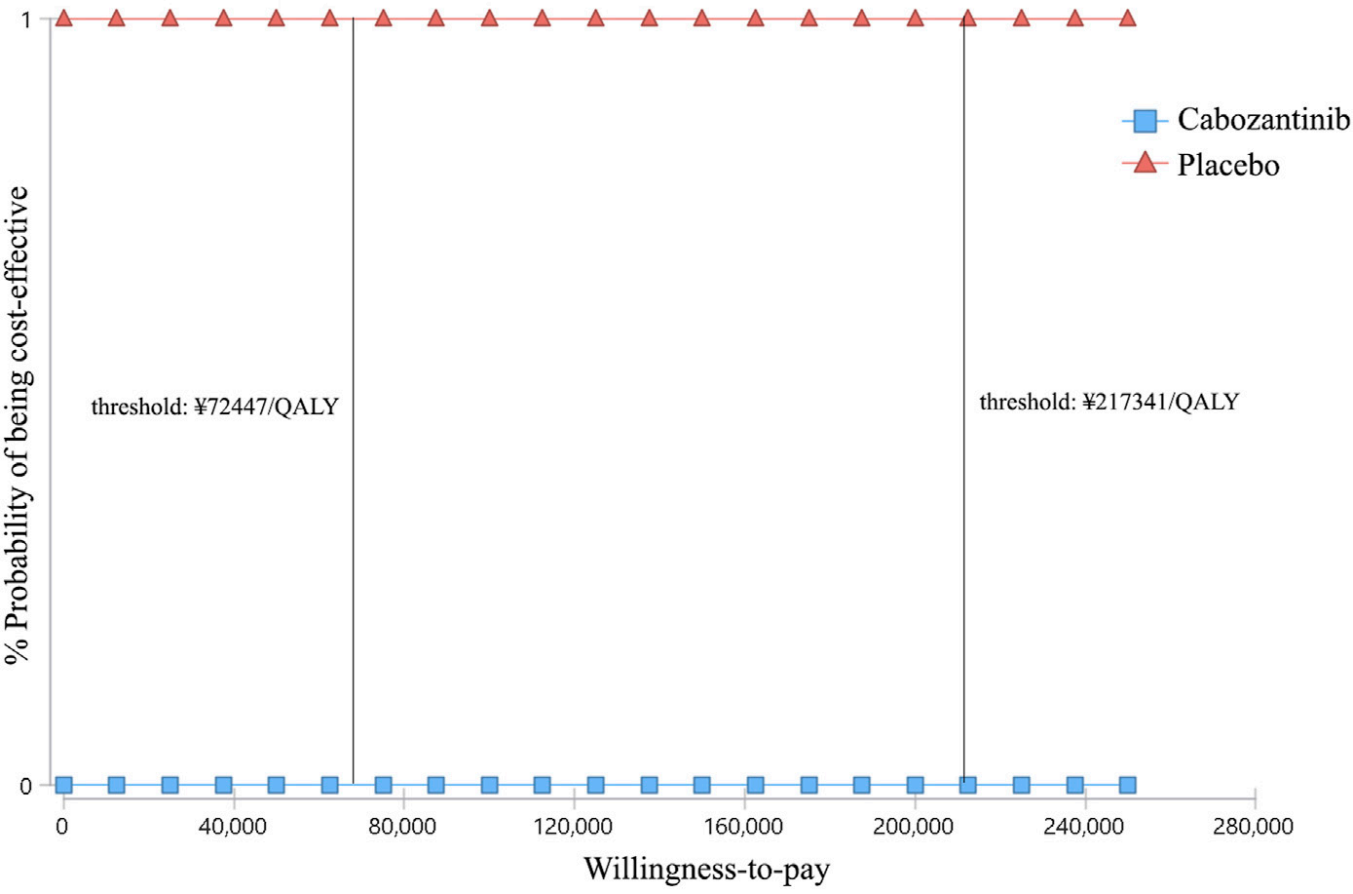
Cost-effectiveness acceptability curves of cabozantinib versus placebo for the treatment of radioactive iodine–refractory differentiated thyroid cancer. The square shapes indicate the probability of cabozantinib being cost-effective when compared with placebo under different willingness-to-pay thresholds.

At the WTP threshold of ¥2,17,341/QALY, apatinib had a 100% of probability being cost-effective when compared with the placebo (Figure 4). However, when compared cabozantinib with placebo, the probability of being cost-effective was 0 (Figure 5). When the unit price of cabozantinib is reduced to ¥5.87/mg (39% of its current price), the likelihood of cost-effectiveness of cabozantinib would increase to 50%.

## Discussion

The unmet demand for treating RAIR-DTC and lacking precise economic evaluation of apatinib and cabozantinib are the motivations of the current study. Furthermore, China has the largest population around the world with fast increasing demands for limited healthcare resources. It has driven the policymakers toward a data-driven and evidence-supporting healthcare system under China’s national health strategy (Tan et al., 2017). We conducted this study to evaluate the cost-effectiveness of apatinib and cabozantinib for the treatment of RAIR-DTC, aiming to provide some useful economic information for their clinical application in the future. According to the results, the ICER at base case estimate for apatinib vs. placebo was ¥93,460/QALY and for cabozantinib vs. placebo was ¥4,52,325/QALY. Both one-way and probabilistic sensitivity analyses proved the robustness of the models. These results suggested that apatinib was cost-effective and cabozantinib might not be cost-effective under the current Chinese healthcare system.

One-way sensitivity analysis showed that the price of drugs was among the most influential factors affecting the final results. However, when the prices varied in their range, the conclusion remained unchanged. Apatinib is marketed in mainland China for the treatment of different cancers including metastatic gastric carcinoma, metastatic breast cancer, advanced hepatocellular carcinoma, and so on (Wang et al., 2018). It has been on the list of basic medical insurance in China with a relatively low price (Ministry of Human Resources and, 2021). The cost-effectiveness of apatinib for the treatment of gastric cancer in China has also been proved (Bai et al., 2017; Liao et al., 2019). Conversely, the cabozantinib is not available in the mainland Chinese market now. Like some other cost-effectiveness investigations (Chen et al., 2021), we used the Hongkong price in our study as many mainlanders would go to Hongkong for some unavailable drugs due to the lower prices and easy accessibility. Probabilistic sensitivity analysis showed that cabozantinib had a 50% likelihood to be cost-effective when its price dropped to ¥5.87/mg, near one-third of its current price. It suggests that a reduction of price might be warranted to enhance the feasibility of using the regimen as a preferred treatment in China. Efforts have been made by the Chinese government by launching the centralized drugs procurement program and the price of many anti-cancer drugs had dropped evidently when they entered the procurement list (State Council of China, 2021).

In this study, we did not compare the apatinib and cabozantinib directly in a single model. Instead, we compared apatinib vs. placebo and cabozantinib vs. placebo in two separate models. Even though a previous study has compared lenvatinib, sorafenib, and placebo directly based on two separate clinical trials, it is one of the major limitations of this study which has been acknowledged by the authors because they were unable to determine the actual compared outcomes without a head-to-head trial (Wilson et al., 2017). In our study, it is risky to put these treatment arms in a single model since the REALITY trial was conducted in China alone while the COSMIC-311 trial was conducted internationally within multiple countries. Moreover, patients in the REALITY trial had a median follow-up duration of 18.1 months while those in the COSMIC-311 trial were only followed for a median of 6.2 months. Future studies comparing the apatinib and cabozantinib directly might be needed.

This analysis has several strengths worth highlighting. First, as far as we know, this is the first modeling study that assessed the economic outcomes of apatinib and cabozantinib for the treatment of RAIR-DTC. The PFS and OS data from the latest randomized phase 3 clinical trials were incorporated into our model. Second, based on the results of sensitivity analysis, we provided a reference for the listing of cabozantinib in China in the future. Third, this study was conducted according to the guidelines of the Consolidated Health Economic Evaluation Reporting Standards (Husereau et al., 2013). The important elements of cost-effectiveness analysis including the target population, study perspective, comparator, time horizon, assumptions, effectiveness measurement, and so on have been clearly described.

However, the limitations of this study should be noted. First, as we have mentioned above, we are not able to compare the apatinib and cabozantinib directly due to the lack of a head-to-head trial comparing these treatments directly. Furthermore, we did not have access to individual patients’ data and the health outcomes were assumed by the fitting of parametric distributions to the reported PFS and OS curves. Though this method has been used widely among oncology studies, extrapolation of Kaplan-Meier curves from median follow-ups of 18.1 and 6.2 months to a 10-year time horizon would inevitably lead to uncertainty when compared to the real clinical settings. Third, unlike the REALITY trial that was conducted solely in China, the COSMIC-311 trial was conducted in 25 countries and Asians accounted for only 18.2% of all participants. It might not reflect the treatment effect of the Chinese population specifically. Fourth, the utility data were not reported by the two clinical trials and we assumed they were similar to the previous study which estimated the utilities for RAIR-DTC among United Kingdom population (Fordham et al., 2015). However, this is not unprecedented and sensitivity analyses have accounted for the difference.

In conclusion, apatinib is a cost-effective treatment of RAIR-DTC when compared with placebo in China. However, based on the current evidence, cabozantinib might not be cost-effective from the Chinese healthcare system perspective and a reduction of price is warranted.

## Data Availability

The raw data supporting the conclusion of this article will be made available by the authors, without undue reservation.
